# DEC205-DC targeted DNA vaccine against CX3CR1 protects against atherogenesis in mice

**DOI:** 10.1371/journal.pone.0195657

**Published:** 2018-04-11

**Authors:** Jimmy Jianheng Zhou, Yuan Min Wang, Vincent W. S. Lee, Geoff Yu Zhang, Heather Medbury, Helen Williams, Ya Wang, Thian Kui Tan, David C. H. Harris, Stephen I. Alexander, Anne M. Durkan

**Affiliations:** 1 Centre for Kidney Research, Children’s Hospital at Westmead, Westmead, NSW, Australia; 2 University of Sydney, Sydney, NSW, Australia; 3 Centre for Transplantation and Renal Research, University of Sydney at Westmead Institute of Medical Research, Westmead, NSW, Australia; 4 Vascular Biology Research Centre, Surgery, University of Sydney, Westmead Hospital, University of Sydney, Westmead, NSW, Australia; Max Delbruck Centrum fur Molekulare Medizin Berlin Buch, GERMANY

## Abstract

Studies disrupting the chemokine pathway CX3CL1 (fractalkine)/ CX3CR1 have shown decreased atherosclerosis in animal models but the techniques used to interrupt the pathway have not been easily translatable into human trials. DNA vaccination potentially overcomes the translational difficulties. We evaluated the effect of a DNA vaccine, targeted to CX3CR1, on atherosclerosis in a murine model and examined possible mechanisms of action. DNA vaccination against CX3CR1, enhanced by dendritic cell targeting using DEC-205 single chain variable region fragment (scFv), was performed in 8 week old ApoE-/- mice, fed a normal chow diet. High levels of anti-CX3CR1 antibodies were induced in vaccinated mice. There were no apparent adverse reactions to the vaccine. Arterial vessels of 34 week old mice were examined histologically for atherosclerotic plaque size, macrophage infiltration, smooth muscle cell infiltration and lipid deposition. Vaccinated mice had significantly reduced atherosclerotic plaque in the brachiocephalic artery. There was less macrophage infiltration but no significant change to the macrophage phenotype in the plaques. There was less lipid deposition in the lesions, but there was no effect on smooth muscle cell migration. Targeted DNA vaccination to CX3CR1 was well tolerated, induced a strong immune response and resulted in attenuated atherosclerotic lesions with reduced macrophage infiltration. DNA vaccination against chemokine pathways potentially offers a potential therapeutic option for the treatment of atherosclerosis.

## Introduction

Atherosclerosis is a chronic inflammatory disease characterized by progressive infiltration of monocytes into the endothelium with the formation of plaques containing lipids, leukocytes, smooth muscle cells (SMCs) and inflammatory mediators. These plaques can become unstable and prone to rupture, triggering acute thrombotic vascular events resulting in myocardial infarction, stroke, and sudden cardiac death.

Monocytes play a major role in atherosclerotic plaque development. There are two major subsets of circulating murine monocytes based on the chemokine receptors expressed. Classical (Ly-6C^hi^) monocytes have high levels of CCR2 and low levels of CX3CR1, whereas non-classical (Ly-6C^lo^) monocytes are CCR2 low and CX3CR1 high. Early in atherogenesis CCL2 plays an important role in attracting inflammatory monocytes but crucially these monocytes also require CX3CR1 to enter into the plaque [1]. The infiltrated monocytes mature to give rise to macrophages, some of which accumulate lipid to become foam cells. Macrophages polarize to different phenotypes depending on the stimuli in the microenvironment with the simplistic classification describing M1 macrophages as being pro-inflammatory and M2 macrophages generally being anti-inflammatory [2]. Macrophages can also proliferate within the plaques. In the mouse, chemical depletion of macrophages markedly attenuates atherosclerosis [3]. Since macrophage movement out of the plaque is rare and the continuous accumulation of monocytes in the plaque is associated with an increase in lesion size, targeting monocyte/macrophage influx is an exciting potential therapeutic option for inhibiting disease progression.

Chemokines play an important role in regulating chemotaxis and coordinating leukocyte trafficking and activation during an inflammatory response [3, 4]. Two chemokine pathways appear crucial to macrophage recruitment, CCL2 (and its receptor CCR2) and Fractalkine (CX3CL1) (and its receptor CX3CR1). Other chemokines such as CCL5 may have less significant roles. CCL2/CCR2 and CX3CL1/CX3CR1 contribute significantly to the recruitment and stimulation of monocytes/macrophages during the pathogenesis of atherosclerosis, and are expressed in early and advanced atherosclerotic lesions in humans and mice [5–7]. CX3CL1 has a dual role, functioning as both a chemokine and an adhesion molecule for monocytes [8]. Furthermore, CX3CL1 expression on endothelial cells triggers the activation and adhesion of platelets, a process that marks the initiation of atheroma formation [9]. Activated platelets degranulate and release P-selectin promoting direct platelet–leukocyte interactions and subsequent leukocyte recruitment and transmigration [10]. CX3CL1 is also chemotactic for SMCs that infiltrate atheromatous plaques. Atherosclerosis is ameliorated in ApoE-/- mice in which the CX3CL1/CX3CR1 pathway has been genetically deleted [11–13] or pharmacologically blocked [14]^,^[15]. The methods used to date to inhibit chemokine pathways have been limited by the difficulty in translating interventions into viable therapeutic options for human studies.

DNA vaccination delivers plasmid DNA encoding the target antigen and induces specific cellular and antibody responses; however the efficacy is often limited particularly against self-antigens [16–18]. The effect of DNA vaccination can be enhanced by targeting the plasmid to dendritic cells by combining it with a single-chain Fv antibody (scFv) specific for the dendritic cell (DC)-restricted antigen-uptake receptor DEC205. Using this technique, strong and protective immunity in mice has been achieved with substantially increased antibody levels and cellular responses in comparison with non-DC target vaccination. The ability to break self-tolerance has also been demonstrated against a number of self-molecules, such as CD40, but has frequently required secondary priming with a peptide known as “prime-boost”[19–21]. We have previously shown functional blocking antibodies to CX3CR1 are induced by DNA vaccination in *in vitro* transmigration assays[22].

To assess the effect of blocking the CX3CL1/CX3CR1 pathway on atherogenesis we developed a DC-targeted DNA vaccination against CX3CR1 that was used in a murine model of atherosclerosis,feeding the mice a normal chow diet to more closely mimic the atherosclerotic lesions found in humans. This vaccination resulted in decreased macrophage recruitment and significant protection from atherosclerosis.

## Materials and methods

### Construction, modification and testing of CX3CR1 DNA vaccines

Vectors DEC205 (pSC-DEC-OLLA) and control vector (pSC-GL117-OLLA) were kindly provided by Dr Godwin Nchinda from The Laboratory of Cellular Physiology and Immunology, Rockefeller University, New York, NY 10065–6399, USA[23]. The pSC-DEC-OLLA vector (scDEC) is a modified pcDNA3.1 vector that contains the gene encoding single-chain antibody specific for mouse dendritic cell antigen receptor DEC205 [23]. Mouse CX3CR1 cDNA was obtained by reverse transcription using SuperScript® II Reverse Transcriptase (Life Technologies, USA) was amplified from C57/BL6 mouse kidney total RNA extracted using trizol reagents (Gibco BRL, Life Technologies, NY, USA). The mouse CX3CR1 cDNA was amplified by PCR using the specific primers, mouse CX3CR1: CX3CR1-NTerm-FW: 5’- CTCACCATGTCCACCTCCTTTCGAGCGGCCGCCTCACCATGTCCACCTCCTT-3’, CX3CR1-CTerm-ReV: 5’-GGAGACCCCTTCAGAGCAGCTGACCGCGGGGAGACCCCTTCAGAGCAG-3’. The PCR conditions were 95°C for 5 min (1 cycle), 95°C for 45 s, 60°C for 45 s, and 72°C for 1 min (35 cycles) with the final cycle at 72°C for 7 min. The CX3CR1 PCR products were ligated separately by T4 DNA ligase (Promega) into DEC205 (DEC-CX3CR1) and control vector (Con-CX3CR1) plasmids to make the two sets of DNA vaccines. The ligated CX3CR1 constructs were cloned into JM109 competent *E*.*coli* cells (Promega) and verified by DNA sequencing after cloning using specific primers, 5’-GCGAATGAATTGGGACCT-3’ and 5’-CTTCTGAGATGAGTTTTTGTTCG. Plasmid DNA was prepared using Qiagen Plasmid Max Kit (Qiagen).

The fusion DNA constructs (DEC-CX3CR1 and Con-CX3CR1) were transiently transfected into CHO cells (ATCC) using Lipofectamine® LTX Reagent (Life Technologies, USA) for immunoblot analysis to verify their expression. One day prior to transfection, CHO cells were grown and maintained at 80% confluence and cells were then diluted to 5X10^4^ /ml of serum free Gibco® Dulbecco's Modified Eagle's medium (DMEM, Life Technologies). DNA plasmids were diluted in serum free Opti-MEM® I Medium (4ug/ml, Life Technologies, USA) and mixed with pre-diluted Lipofectamine® LTX Reagent for 30mins at RT before adding to the CHO cells and incubating at 37°C for 24–48 hours. Total cell supernatant were collected for the incoming western blot analysis on CX3CR1 expressing NIH 3T3 cell lysate.

### DNA vaccination

Six weeks old male C57/BL6 ApoE-/- mice (stock number 002052) were purchased from the Animal Resources Centre in Perth, Australia and maintained under standard sterile conditions in the Department of Animal Care at Westmead Hospital, NSW. These mice were generated by backcrossing the Apoetm1Unc mutation 10 times to C57BL/6J mice. This study was specifically approved by the Animal Ethics Committee of Sydney West Area Health Service. All mice were fed with normal diet. Mice were divided into four groups: DEC-CX3CR1 vaccinated (n = 6), Con-CX3CR1 vaccinated (n = 6), adjuvant only control (n = 6), and non-vaccinated control (n = 6). Six weeks old male wildtype C57/BL6 mice (n = 6) were also obtained from the Animal Resources Centre in Perth, Australia, they were fed with normal chow and maintained under standard sterile conditions in the Department of Animal Care at Westmead Hospital, NSW. The Health and well-being of mice were monitored 3 times every week.

All mice except the non-vaccinated controls were pretreated with 0.5% bupivacaine (1 /g body wt; Sigma, St. Louis, MO) by intramuscular injection into the tibialis anterior muscle two weeks before plasmid DNA vaccination. Plasmid DNA (50 μg) was injected at the same site as bupivacaine three times at two weekly intervals, into the mice from the vaccinated groups. The mice were anaesthetised with Isoflurane inhalation (Sigma), the isoflurane vaporizer were adjusted to 3.5% for induction and approximately 1.5% for maintenance, the oxygen flowmeter was adjusted to approximately 0.9 L/min. The efficiency of DNA vaccination was enhanced by electroporation system (BTX Harvard Apparatus). One week after the third DNA vaccination, mice from the DEC-CX3CR1 and Con-CX3CR1 vaccinated groups were immunized with a prime boost of CX3CR1 peptide mixture (100 μg/mouse, mixed in Freund's Complete Adjuvant, GeneTex, Irvine, CA) and Poly IC (50 μg/mouse). Mice from the non-vaccinated and adjuvant only control groups were injected with saline only. Blood was collected by tail bleeding from all mice at 2, 4, and 8 weeks after peptide boost or saline injection, for ELISA. All mice were sacrificed at 34 weeks, by inhalation of CO2 gas at which point, the whole blood was collected by cardiac puncture for ELISA and cholesterol measurement. The aortas and brachiocephalic arteries were excised, fixed in OCT tissue freezing medium (Leica systems) and frozen for histology and immunostaining analysis.

### Serum antibody titers

Serum anti-CX3CR1 antibody titers were evaluated by ELISA assay for all groups of mice. A 96 well Immuno ELISA microtiter plate (NUNC, *in vitro* Technology, Australia) was coated with CX3CR1 peptide (Genetex) at a concentration of 1 μg/well in 100 μl coating buffer and incubated overnight. Sample mouse sera were diluted 50 and 500 times and added to the coated ELISA plate. Wild type C57/BL6 mouse serum was used as a negative control. Goat anti-mouse IgG alkaline phosphatase conjugated Ab (Sigma) and p-Nitro-phenol phosphate (Sigma) as substrate were added sequentially for the Ab titer analysis. All samples and controls were added in duplicate to the plates. Absorbance was read at 450 nm on an ELISA plate reader (Multiskan Ascent, Pathtech, Australia).

### Quantification of atherosclerosis and lipid content

OCT-embedded frozen brachiocephalic artery and aortic arch samples were sectioned proximal to distal at 5 μm intervals. Haemotoxylin and Eosin (H&E) stained samples were used to visualize plaque size. Frozen sections of brachiocephalic arteries were fixed in ice-cold acetone (10 min) and washed with running tap water for 1 minute. Sections were re-hydrated in 2 changes of absolute alcohol, 5 minutes each, followed by 2 mins in 95% alcohol and another 2 mins in 70% alcohol. Sections were washed briefly in distilled water and stain in Harris hematoxylin solution for 10 minutes. Sections were washed in running tap water for 5 minutes and differentiated in 1% acid alcohol for 30 seconds. Sections were washed in running tap water for 1 minute and then Blued in 0.2% ammonia water for 1 minute. Sections were then washed in running tap water for 5 minutes and rinsed in 95% alcohol for 10 dips. Sections were counterstained in eosin solution for 1 minute. Sections were dehydrated in graded ethyl ethanol (70%, 95% and 100% 2 changes each at 5 minutes) and two changes of xylene, 5 minutes each and then permanently mounted with VectaMount mounting medium (Vector Laboratories).

OCT-embedded frozen brachiocephalic artery samples were sectioned proximal to distal at 5 μm intervals and lipids were detected with Oil Red O. Frozen sections of brachiocephalic arteries were fixed in ice-cold acetone (10 min) and washed with running tap water for 1 minute. Sections were rinsed with 60% isopropanol and stained with freshly prepared Oil Red O working solution 15 mins and then rinsed with 60% isopropanol. Sections were stained for nuclei with alum haematoxylin in 5 dips and rinsed with distilled water. Sections were mounted in glycerine jelly. At least 3 sections per mouse each 100 μm apart were inspected for each histostaining.

Plaque size and lipid content were quantified using *Aperio Imagescope* software, images of the stained sections were scanned at original size and 20X of magnification for analysis.

### Quantification of macrophage and alpha-smooth muscle cell infiltration

Frozen sections of brachiocephalic arteries were fixed in ice-cold acetone (10 min) and washed with Tris-*Buffered* Saline and Tween 20 (TBST). Peroxidases were quenched by Peroxidase-Blocking Solution (Dako) for 10 min. Sections were washed and blocked with Protein Block Serum-Free solution (Dako) for 10 min. Sections were incubated with the primary antibodies for 60 min (rat anti-mouse MOMA-2, Bio-rad, final concentration: 0.01mg/ml, rabbit anti-mouse α-Smooth Muscle Actin, Abcam, final concentration: 0.2 mg/ml, rat anti-mouse CD86, Biolegend, final concentration: 0.01mg/ml and rabbit anti-mouse CD206 (H-300), Santa Cruz final concentration: 0.01mg/ml). Sections were washed and incubated with horseradish peroxidase (HRP)-conjugated secondary antibody for 30 min (Rat-on-Mouse HRP-Polymer Kit, Biocare Medical, for MOMA-2 and CD86; Labeled Polymer-HRP EnVision™ anti-rabbit, DAKO, for α-SMA and CD206). After washing, sections were developed with incubation with 3, 3’-Diaminobenzidine tetra-hydrochloride (DAB, DAKO) and sections were counterstained with hematoxylin for 1 min followed by bluing within continuous water for 20 minutes. Sections were dehydrated in graded ethyl ethanol (70%, 80% and 100%) and two dips of histolene, and then permanently mounted with VectaMount mounting medium (Vector Laboratories). The macrophage, smooth muscle cell and M1 and M2 contents were quantified in three equidistant stained sections (100 μm apart) using *Aperio Imagescope* software. Images of the stained sections were scanned at original size and 20X of magnification for analysis.

### Serum cholesterol analysis

At the time of euthanasia, whole blood was drawn by cardiac puncture. Serum was obtained by centrifugation for 10 minutes at 2,000 x g 4°C. Serum cholesterol levels were measured with a commercial cholesterol kit (Wako Diagnostics) as per the manufacturer’s instructions. Serum was also obtained from Wildtype C57/BL6 mice (n = 6) and analysed as control.

### Serum cytokine analysis

The BD Cytometric Bead Array Mouse Inflammatory Kit (BD Bioscience) was used to measure IL-6, IL-12, CCL2, TNF-α, TFN-γ and IL-10 serum proteins, following the manufacturer’s instructions (BD Bioscience). In brief, all serum samples were pre-diluted to the manufacturer’s recommended concentration with distil water and mixed with the prepared capture beads at a 1:1 ratio (50ul each) before incubation at 37°C for 2 hours without exposure to light. Samples were washed with wash buffer and centrifuged at 300g and then resuspended before measurement by FACS Calibur flow cytometry. The data were analyzed by FCAP Array software (BD).

### Statistical analysis

Results are expressed as the group mean ± SEM. Statistical analysis was performed using one-way analysis of variance (ANOVA) for multiple comparisons using GraphPad Prism software. Two group differences were analyzed by Student’s *t-*test, with a *p* value (two-tailed) less than 0.05 considered statistically significant.

## Results

### Construction and expression of scDEC and scControl DNA vaccines

**Fig 1:**
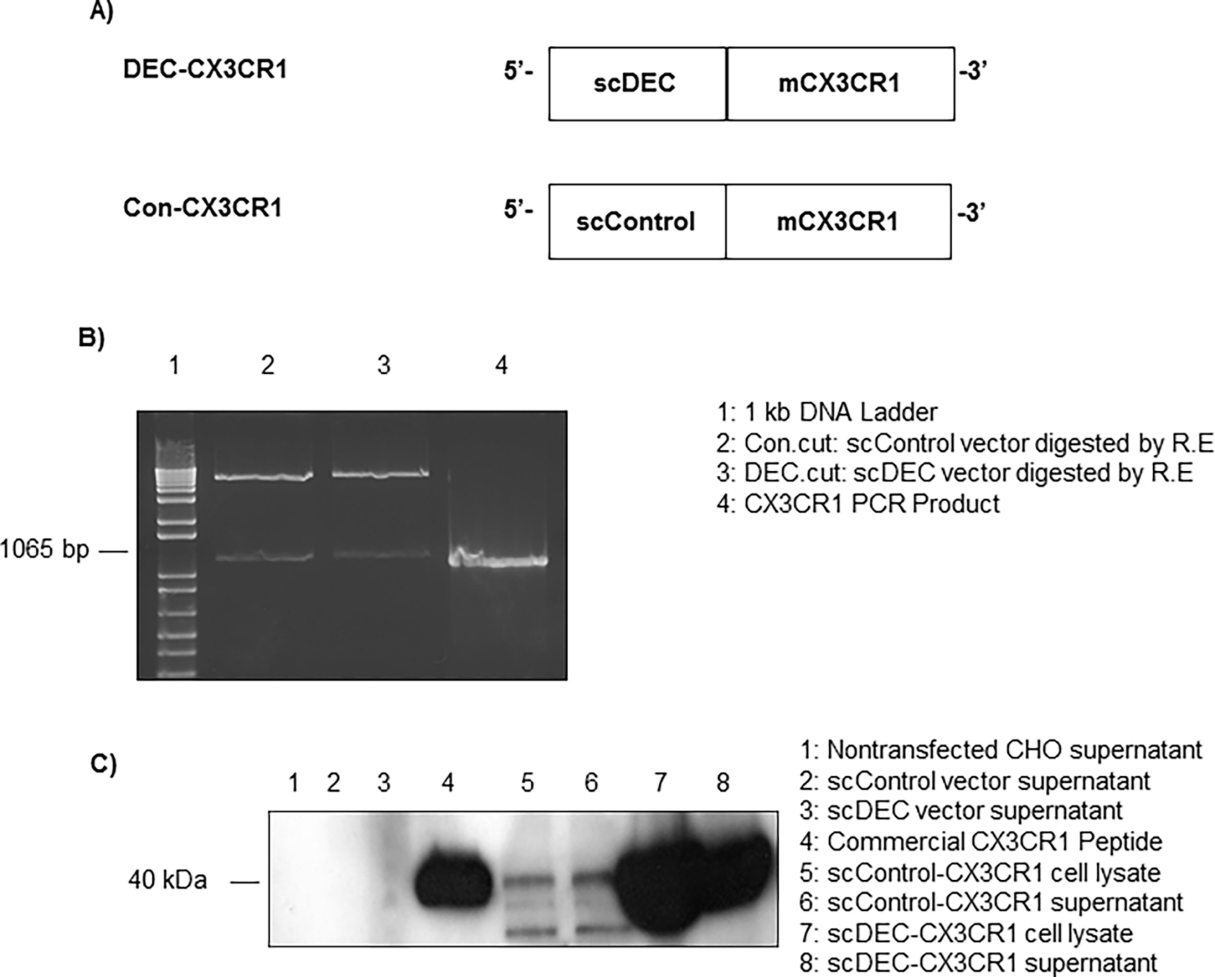
Construct with in vitro and in vivo expression of DEC-CX3CR1 and Con-CX3CR1 DNA vaccines. (A) Open reading frames for mouse CX3CR1 extracellular domain were fused in frame to the C-terminus of scDEC or scControl vector. (B) The CX3CR1 PCR products were ligated and cloned separately into DEC205 (DEC-CX3CR1) and control vector (Con-CX3CR1), in agarose gels and visualized under UV using gel-doc 1000 (BIO-RAD, Australia, two separate bands as indicated in DEC.cut and Con.cut after restriction digestion). (C) Plasmid DNAs (scControl-CX3CR1, scDEC-CX3CR1, scControl vector, and scDEC vector) were transiently transfected into CHO cells and 24 hours post transfection total cell supernatant were subjected to western blot.

We generated fusion DNA constructs with CX3CR1 genes fused in frame to the C-terminus of scDEC as shown in Fig 1A. The non-DC-targeted CX3CR1 DNA vaccines (Con-CX3CR1) were generated with the use of pSC-GL117-OLLA (scControl) vector instead of scDEC vector (Fig 1B). The protein expression of the DEC-CX3CR1 and Con-CX3CR1 were confirmed by Western blot of CHO cell line transfected supernatants (Fig 1C).

### CX3CR1 DNA vaccination induces anti-CX3CR1 antibody response

**Fig 2:**
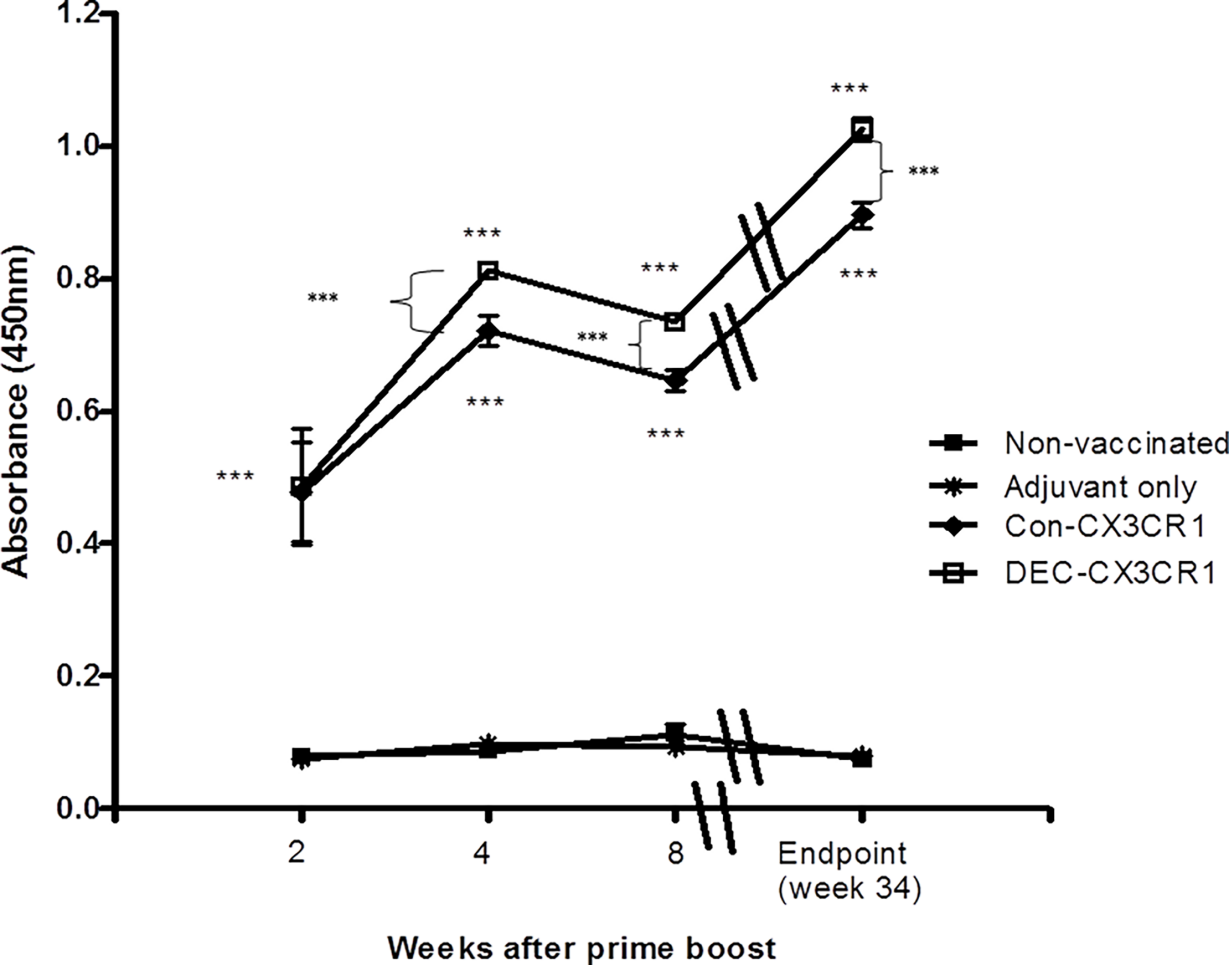
CX3CR1 DNA vaccination induced significant humoral responses. Anti-CX3CR1 antibody levels in serum were measured by ELISA at 2, 4 and 8 weeks after prime boost of CX3CR1 peptide and at the endpoint when mice were sacrificed (34 weeks of age). Absorbance was read at 450nm corrected against background reading at 570nm. DEC-CX3CR1 vaccine induces significantly higher serum anti-CX3CR1 antibody level as compared to Con-CX3CR1 vaccination at 4 and 8 weeks after prime boost and at the endpoint. Both DEC-CX3CR1 and Con-CX3CR1 vaccine induces significantly higher serum anti-CX3CR1 antibody as compared to adjuvant only and non-vaccinated control group at all time points (*** p< 0.001). Represented results from 6 mice in each group.

To determine whether DNA vaccines could induce a strong humoral response, we measured serum anti-CX3CR1 antibody level using ELISA. Both the anti-CX3CR1 vaccines stimulated a marked response compared to controls (p<0.001 for both vaccines) but on direct comparison this was greater in the DEC-CX3CR1 vaccinated mice beyond 2 weeks post CX3CR1 prime boost (p<0.001) (Fig 2). There were no apparent adverse effects of the vaccine.

### CX3CR1 DNA vaccination ameliorates atherosclerosis

**Fig 3:**
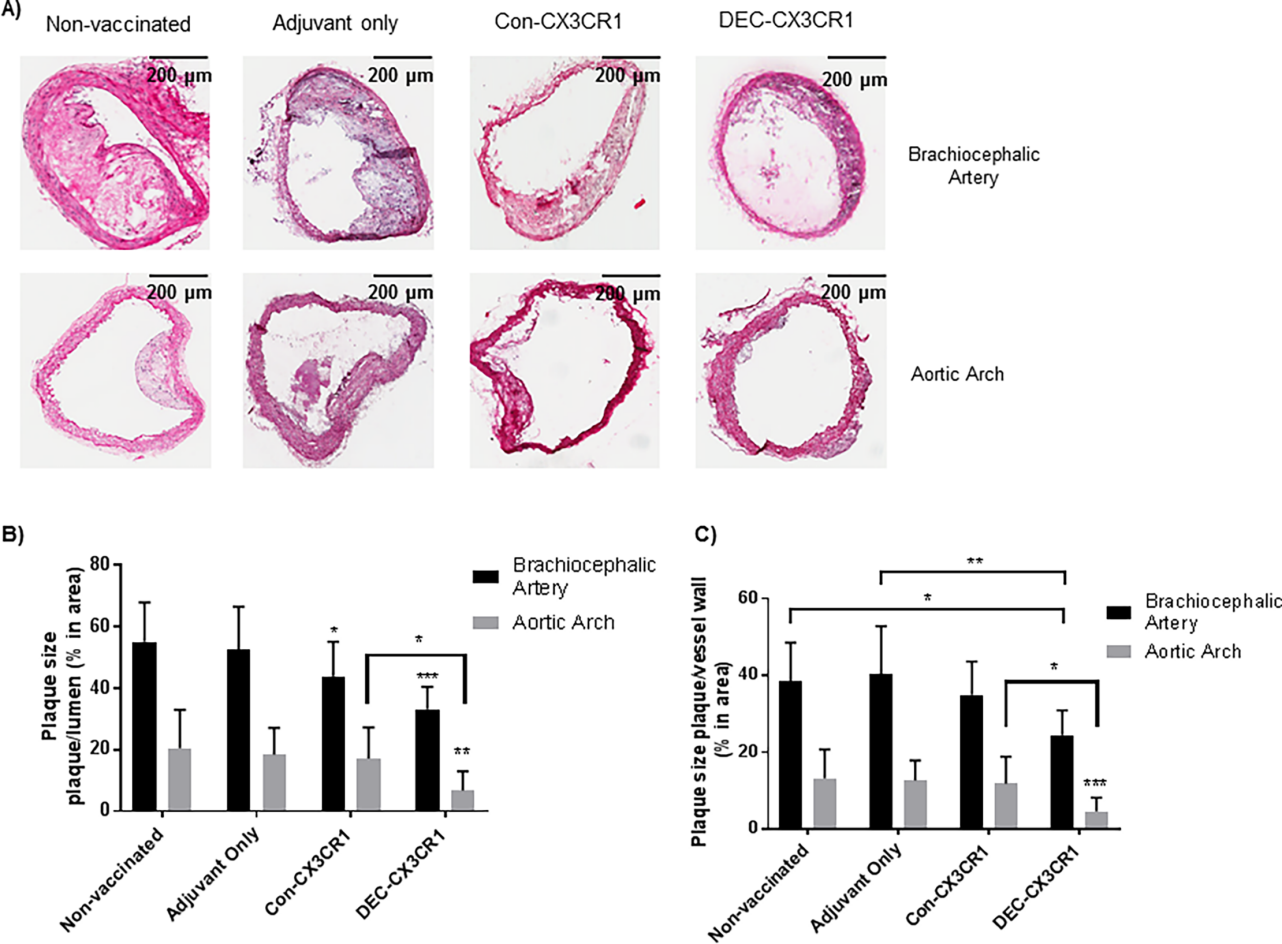
CX3CR1 DNA vaccination reduced plaque formation in brachiocephalic arteries and aortic arch of atherosclerotic ApoE-/- mice. (A) Representative images of atherosclerotic lesions from brachiocephalic arteries with Haemotoxylin & Eosin staining. (B) Quantitative analysis showing that there were significant reductions in plaque size (area of plaque/Lumen) in brachiocephalic arteries of both DEC-CX3CR1 vaccinated mice and Con-CX3CR1 vaccinated mice in comparison to adjuvant only and non-vaccinated control mice (*** p< 0.001;*p<0.05 respectively). (C) Quantitative analysis showing that there were significant reductions in plaque size (area of plaque/vessel) in brachiocephalic arteries of DEC-CX3CR1 vaccinated mice in comparison to adjuvant only and non-vaccinated control (**p< 0.01;*p<0.05 respectively).

To test the effect of vaccination on atherosclerosis we examined the brachiocephalic arteries and aortic arches from 34 week-old ApoE-/- mice (Fig 3) as these arteries consistently had macroscopically visible plaque formation in the control animals. DEC-CX3CR1 vaccinated mice exhibited a significant reduction in plaque size as compared to non-vaccinated control mice (p<0.001, 35% reduction in plaque area/luminal area, p<0.05, 24% reduction in plaque area/vessel area) and mice with adjuvant only (p<0.001, 35% reduction in plaque area/luminal area, p<0.01, 33% reduction in plaque area/vessel area). A reduction in plaque development was also observed in the brachiocephalic artery from the Con-CX3CR1 vaccinated mice as compared to mice with adjuvant only (p<0.05, 22% reduction in plaque area/luminal area,) and non-vaccinated control mice (p<0.05, 24% reduction in plaque area/luminal area). Despite the better humoral response in the DEC-CX3CR1 vaccinated mice, compared to Con-CX3CR1 mice, there was no significant difference in plaque size between the two groups.

### Inhibition of lipid deposition in plaques by CX3CR1 vaccination

**Fig 4:**
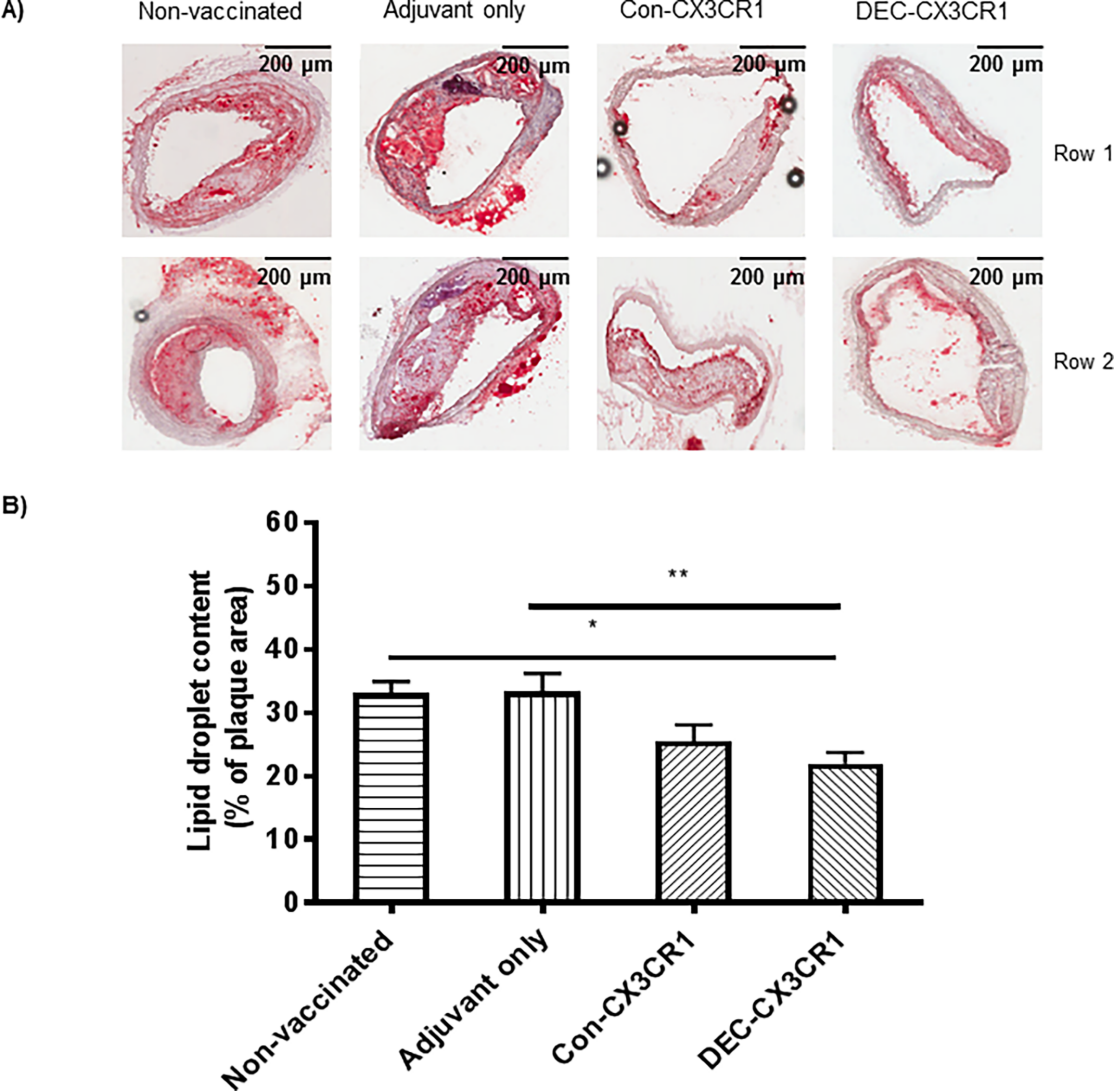
CX3CR1 DNA vaccination reduced lipid deposition in plaques in brachiocephalic arteries of atherosclerotic ApoE-/- mice. (A) Representative images of atherosclerotic lesions from brachiocephalic arteries stained with Oil Red O. (B) Quantitative analysis showing that lipid deposition is significantly reduced in DEC-CX3CR1 vaccinated mice as compared to adjuvant only and non-vaccinated control mice (**p< 0.01;*p<0.05 respectively). Each row represents a single experimental mouse within a group.

Atherosclerotic plaques are made up mainly of lipid laden macrophages and inflammatory infiltrates. To examine the effect of the vaccination on the lipid content of the plaque, we stained sections with oil red O, (Fig 4). DEC-CX3CR1 vaccinated mice exhibited a significant reduction in fat deposition in brachiocephalic arteries as compared to non-vaccinated control mice (p<0.05, 34% reduction) and mice with adjuvant only (p<0.01, 36% reduction).

### Inhibition of macrophage infiltration in plaques by CX3CR1 vaccination

**Fig 5:**
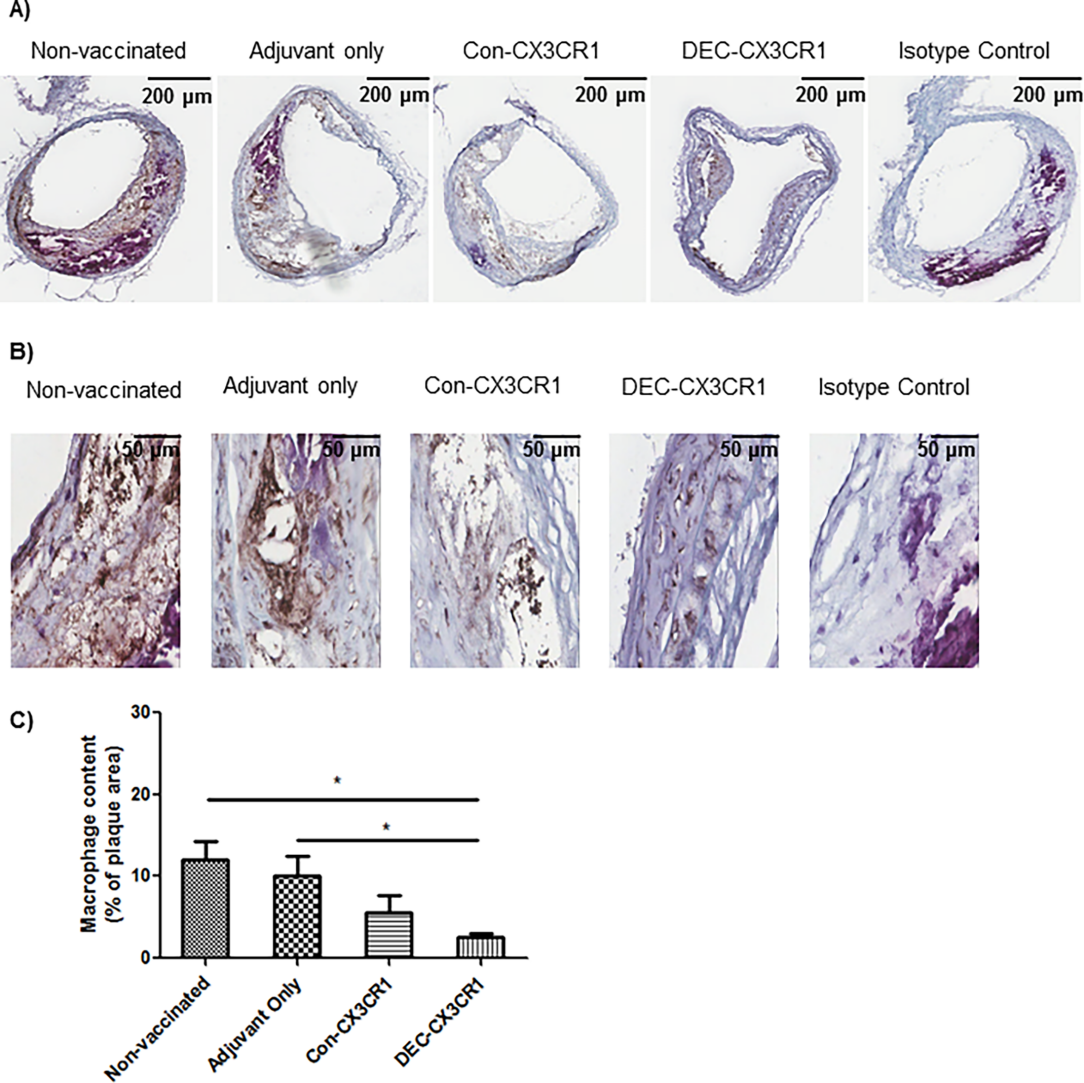
CX3CR1 DNA vaccination reduced macrophage infiltration in plaques in brachiocephalic arteries of atherosclerotic ApoE-/- mice. (A)&(B) Representative images (2X/40X magnification, respectively) of atherosclerotic lesions from brachiocephalic arteries stained with MOMA-2. (C) Quantitative analysis showing that macrophage infiltration is significantly reduced in DEC-CX3CR1 and Con-CX3CR1vaccinated mice as compared to non-vaccinated control and adjuvant only mice (*p<0.05).

Since CX3CL1 is a known chemoattractant for monocytes and macrophages we next examined whether DNA vaccination against CX3CR1 resulted in lower numbers of macrophages in the plaque. We found significantly less macrophage accumulation in the brachiocephalic artery lesions of the DEC-CX3CR1 vaccinated mice compared with control groups (Fig 5). DEC-CX3CR1 vaccinated mice exhibited a marked decrease in macrophage accumulation within the plaques as compared to both non-vaccinated control mice (5 folds reduction, p<0.05) and mice with adjuvant only (3 folds reduction, p<0.05). Con-CX3CR1 vaccinated mice exhibited a non-significant reduction in macrophage accumulation as compared to non-vaccinated control mice and mice with adjuvant only.

### M1/M2 macrophage cell infiltration

**Fig 6:**
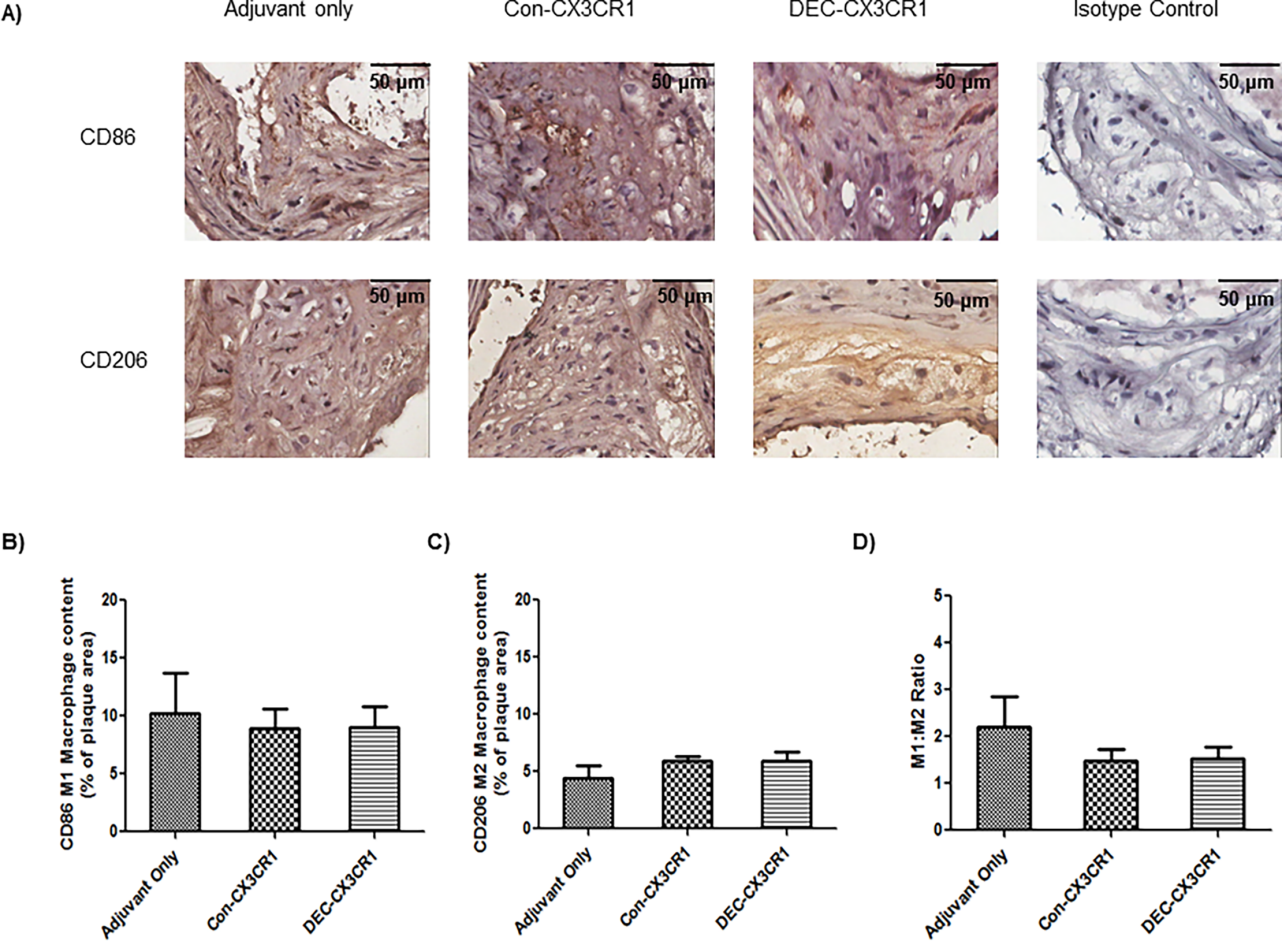
CX3CR1 DNA vaccination did not alter the macrophage phenotype in plaques in brachiocephalic arteries. (A) Representative images (40X magnification) of atherosclerotic lesions from brachiocephalic arteries stained with CD86 and CD206. (B)&(C). Quantitative analysis showing that M1 and M2 macrophage infiltration is not significantly different in DEC-CX3CR1 and Con-CX3CR1 vaccinated mice as compared to adjuvant only mice. (D) Quantitative analysis showing that the M1/M2 macrophage ratio is not significantly different in DEC-CX3CR1 and Con-CX3CR1 vaccinated mice as compared to adjuvant only mice.

Macrophages can be further subtyped based on surface markers and biological function, with the M1 inflammatory phenotype predominating in atherosclerosis. We therefore examined if DNA vaccination against CX3CR1 resulted in altered macrophage phenotype within the atheromatous plaques. Although both DEC-CX3CR1 and Con-CX3CR1 vaccinated mice exhibited a minor increase in M2 macrophage accumulation in brachiocephalic artery lesions, the difference (133% and 118% compared to Adjuvant only) was not significant (Fig 6). It has been argued that the ratio of M1/M2 is more important than the absolute numbers of macrophages as this may influence the biological activity[2]. The M1/M2 ratio was 2.20 for mice with Adjuvant only, 1.47 for Con-CX3CR1 vaccinated mice, and 1.52 for DEC-CX3CR1 vaccinated mice. These differences were not significant.

### Smooth muscle cell accumulation

**Fig 7:**
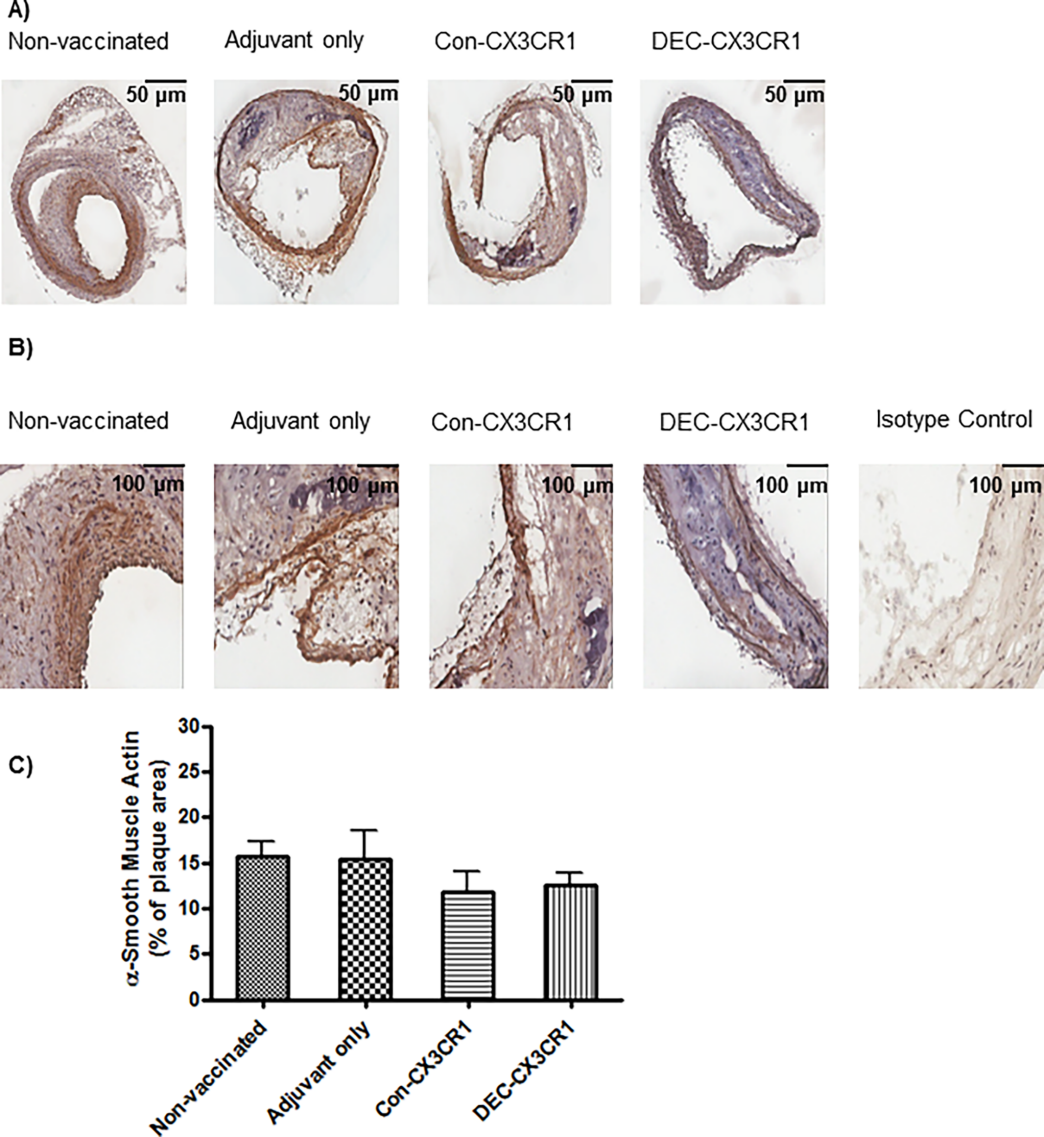
CX3CR1 DNA vaccination did not reduce smooth muscle cell infiltration in plaques in brachiocephalic arteries. (A)&(B) Representative images (2X/20X magnification, respectively) of atherosclerotic lesions from brachiocephalic arteries stained with α-Smooth Muscle Actin. (C) Quantitative analysis showing that smooth muscle cell infiltration was not reduced in DEC-CX3CR1 and Con-CX3CR1vaccinated mice as compared to adjuvant only and non-vaccinated control mice.

In addition to being a chemoattractant for leucocytes, CX3CL1 is also involved in the migration of smooth muscle cells into atherosclerotic plaques [24]. We therefore examined the role of DNA vaccination against CX3CR1 on smooth muscle cell accumulation in atherosclerotic plaques. We found that DNA vaccination did not alter the smooth muscle cell accumulation in brachiocephalic artery lesions from the vaccinated mice compared with control groups, suggesting the protective effect occurs at the level of the macrophage (Fig 7).

### Total serum cholesterol level

**Fig 8:**
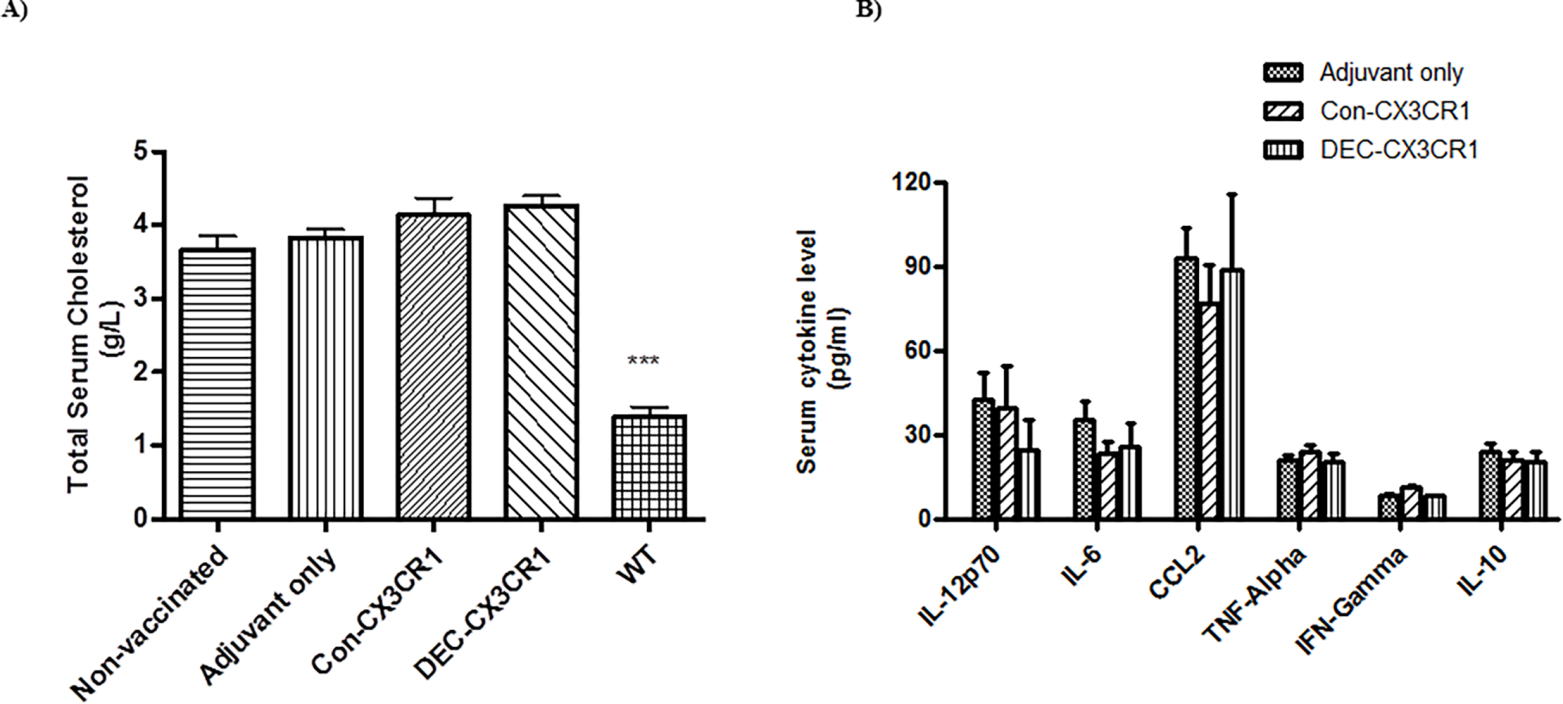
DNA vaccination did not alter the serum cholesterol levels and the cytokine profiles. (A) Serum cholesterol is raised ApoE-/- mice but is not altered by DNA vaccination. Quantitative analysis showing that total serum cholesterol level of the vaccinated mice had no significant differences as compared to the adjuvant only group and non-vaccinated control group. The results represent four independent experiments. (B) Serum IL-6, IL-12, CCL2, TNF-α, TFN-γ or IL-10 levels were not different in the two vaccinated groups compared to adjuvant only.

DNA vaccination did not alter the total serum cholesterol levels in ApoE-/- mice in our study (Fig 8A). All atherosclerotic ApoE-/- mice had similar elevations in serum cholesterol compared to WT C57/BL6 mice. The total serum cholesterol level of the DEC-CX3CR1 vaccinated mice was 4.25g/L, Con-CX3CR1 4.1g/L, adjuvant only controls 3.95g/L, non-vaccinated controls 3.75g/L, and WT C57/BL6 1.5 g/L.

### Serum cytokine protein level

Atherosclerosis is a multifactorial disease process with interactions between various immune and inflammatory pathways. To test if anti-CX3CR1 vaccination had a direct impact on other inflammatory molecules we measured various serum cytokines. CX3CR1 DNA vaccination led to a non-significant reduction in IL-12 and IL-6 (Fig 8B), and it did not change the serum protein levels of IFN-γ and TNF-α. DNA vaccination against CX3CR1 had no effect on the serum levels of the Th2 cytokine IL-10 nor on the major monocyte chemoattractant CCL2, suggesting that these are independent pathways for monocyte/macrophage recruitment.

## Discussion

This study demonstrates that DNA vaccination against CX3CR1 results in decreased atherosclerotic plaque size in a murine model of atherogenesis. This was associated with decreased numbers of macrophages within the plaques and may offer a potential therapeutic alternative or supplemental treatment to those currently available for atherosclerosis.

The role of chemokines and chemokine receptors in atherogenesis is well established however many of the models investigating these pathways have used mice where one or more chemokine or receptor has been genetically deleted. Whilst providing confirmatory information on etiology they are not viable treatment options in humans and we therefore sought to develop a method of blocking the CX3CR1-CX3CL1 pathway that could be further developed for human therapy.

DNA vaccination delivers genes that encode target antigens and allows their expression *in vivo* within host cells in order to induce a desired host immune response[23]. This technique has many beneficial features. DNA vaccines have been shown to be safe and easy to administer, even with repeated doses [25]. Furthermore, they can be prepared on a large scale and with a high purity. However, a major drawback with previous DNA vaccines has been the difficulty encountered in generating a humoral response, particularly when trying to break self-tolerance against self-proteins. Self-proteins are processed and presented in the same way as foreign proteins by recognition by T helper cells after presentation by antigen presenting cells, but, instead of inducing strong immune responses, as seen with foreign antigens, there is often negative selection of induced T cells resulting in tolerance. This is accompanied by an attenuated B cell response. In order to break tolerance we used DC targeting aided by the addition of adjuvants in the plasmid and used Complete Freud Adjuvant (CFA), which stimulates the production of TNF-α in antigen presenting cells[26]. Selective targeting of DNA vaccine to dendritic cells was achieved using DEC205, a dendritic cell antigen. Dendritic cells migrate to secondary lymphoid organs where they present antigens with subsequent activation and augmentation of specific T and B cell responses. Our results demonstrate significantly improved humoral responses and these responses are similar to those reported by our group previously and by other groups implementing a similar technique [20–22]. Other groups have shown that administration of naked DNA vaccinations encoding pro-inflammatory chemokines and receptors can generate antibodies to self-molecules and lead to the breakdown of tolerance to the gene products in arthritis [27]. Various other strategies, such as the use of CFA or electroporation to enhance the efficacy of DNA vaccines by facilitating plasmid entry into target cells, have been developed to enhance the immunogenicity in the host and have had further modifications to allow their use in humans [23, 28–30].

A further advantage of DNA vaccinations is the generation of specific antibodies that appear to block the function of encoded molecules on targeted tissues only. This was demonstrated clearly in our study as there was no depletion of other pathways of the immune system as shown by the cytokine profiling, and by others in previous studies, which is crucial for future clinical studies [16, 22, 31, 32].

Given the potential advantages of DNA vaccination there has been recent interest in its use against a variety of immune targets including co-stimulatory pathways, cytokines and chemokines [18, 33, 34]. Modified DNA vaccination has been proven to be effective in mouse models of infectious diseases, cancers and other autoimmune disorders including multiple sclerosis, experimental autoimmune encephalomyelitis and allergic responses [23, 24, 33, 35–37]. In humans there have been clinical trials of DNA vaccines against the human papilloma virus (HPV) and against HIV combined with cytokines IL-12 and IL-15 as adjuvants [38]. Another clinical trial in chronic Hepatitis C virus (HCV) resulted in detectable antibody and T-cell responses without severe adverse reactions following DNA vaccination [33, 39]. Human clinical trials with DNA vaccinations against a host of other diseases including; melanoma, TB, typhoid fever, hepatitis B and cervical cancer have also been reported, with the vaccines demonstrating efficacy against foreign pathogens.

Our finding of a 35% reduction in atherosclerotic plaque size following DEC-CX3CR1 vaccination is in keeping with interruption of the CX3CL1/CX3CR1 pathway using genetic manipulation or pharmacological blockade [11, 13, 15, 40, 41]. The partial inhibitory effect on the atherosclerosis process is similar to that found in previous studies and results from more than one chemokine/receptor pathway being involved. The chemokines CCL2, CCL5 and CX3CL1/CX3CR1 participate independently in plaque formation [12] and inhibition of more than one pathway may have greater effect than inhibition of a single pathway.

The primary mode of action of CX3CL1/CX3CR1 in atherosclerosis seems to be recruitment of monocytes and accordingly in this model there was a marked reduction in the macrophage content of the plaques. Other models of interruption of CX3CL1/CX3CR1 also reported decreases in macrophage/monocyte content of between 40% and 80%, consistent with our findings [13, 40, 41]. Atherosclerotic plaques typically contain both M1 (classically activated) and M2 (alternatively activated) macrophage phenotypes, with polarization being dependent on the microenvironment. There is an increasing spectrum of macrophage subgroups now recognized and other macrophage subgroups are also present within plaques [2]. M1 macrophages are typically in the more rupture prone shoulder areas of the plaque whilst M2 macrophages are usually found in the cellular areas and areas of neovascularization, contributing to plaque stability [42]. Vaccination against CX3CR1 resulted in a slight but non-significant increase in the M2 phenotype suggesting its primary effect was in limiting macrophage infiltration into the plaque, rather than alteration of the microenvironment and macrophage phenotype. Although we did not specifically look at inflammatory signaling molecules such as NFκB in the vascular tissues, we did measure circulating chemokines as a surrogate of changes in the microenvironment and found no significant differences after vaccination. In accordance, Landsman *et al* argued that CX3CR1 is essential in monocyte/macrophage survival (especially the resident M2 subset) since CX3CR1 deficiency results in reduction of Ly6C^low^ cells [43]. However, another study had demonstrated that CX3CR1 deficiency results in reduction of plaque formation with reduced number of SMC but not macrophages [44], the author argued that this discrepancy may be due to specificity of the model (a transplantation of abdominal aortic segment).

CX3CL1/CX3CR1 can contribute to other features of the atherogenesis process, including the chemoattraction, patrolling of monocytes [45], survival and proliferation of smooth muscle cells [24, 46]. The lack of change in the SMC content suggests that for these cells the CX3CL1/CX3CR1 pathway is redundant and SMC infiltration possibly occurs using another mediator. However a number of studies suggest opposing roles of CX3CL1/CX3CR1 SMC infiltration, Ali *et al* demonstrated that CX3CR1 antagonist (a novel drug AZ1220 targeting CX3CR1) reduced SMC infiltration at porcine model [47], while Kumar *et al* suggested that CX3CL1/CX3CR1 plays a pivotal role on SMC proliferation within plaque neovascularization at murine model [48].

In atherosclerosis other groups have targeted DNA vaccines to vascular endothelial growth factor receptor 2 (VEGFR2) [49, 50], TIE2 (an endothelial marker) [27], and CD99 a leukocyte and endothelial antigen[51] resulting in significantly reduced atherosclerosis and leukocyte infiltration in ApoE-/- mice. This is the first report the use of a DNA-vaccine against a chemokine pathway to attenuate atherosclerosis.

In conclusion, this study shows that modified DEC205-CX3CR1 DNA vaccine shows greater protection in atherosclerotic mice. Vaccination results in the attenuation of macrophage infiltration and an overall decrease in atheromatous plaque size. Therefore blocking the CX3CR1 pathway using DNA vaccination may potentially complement current therapies such as lipid lowering agents and anti-platelet drugs.

## Supporting information

S1 Table Results of readings of ELISA on anti-CX3CR1 antibody levels in serum at 2, 4 and 8 weeks after prime boost of CX3CR1 peptide and at the endpoint. (XLSX)

S2 Table Results of measurements of area (in %) of plaque formation vs lumen and vessel wall in brachiocephalic arteries and aortic arch. (XLSX)

S3 Table Results of measurements of lipid deposition (% in plaque) in brachiocephalic arteries. (XLSX)

S4 Table Results of measurements of macrophage content (% in plaque) in brachiocephalic arteries. (XLSX)

S5 Table Results of measurements of M1 and M2 macrophage content (% in plaque) in brachiocephalic arteries. (XLSX)

S6 Table Results of measurements of α-Smooth Muscle Actin (% in plaque) in brachiocephalic arteries. (XLSX)

S7 Table Results of measurements of total serum cholesterol levels in ApoE-/- mice compared to WT C57/BL6 mice. (XLSX)

S8 Table Results of measurements of Serum IL-6, IL-12, CCL2, TNF-α, TFN-γ or IL-10 levels. (XLSX)
